# Healthcare expenditure of intravitreal anti‐vascular endothelial growth factor inhibitors compared with dexamethasone implant for diabetic macular oedema

**DOI:** 10.1111/aos.15151

**Published:** 2022-04-25

**Authors:** Silvia NW Hertzberg, Morten Carstens Moe, Øystein Kalsnes Jørstad, Beáta Éva Petrovski, Emily Burger, Goran Petrovski

**Affiliations:** ^1^ Department of Ophthalmology, Faculty of Medicine, Center for Eye Research, Oslo University Hospital and Institute for Clinical Medicine University of Oslo Oslo Norway; ^2^ Department of Health Management and Health Economics University of Oslo Oslo Norway; ^3^ Center for Health Decision Science Harvard T. H. Chan School of Public Health Boston MA USA

**Keywords:** Avastin, diabetic macular edema, Eylea, healthcare expenditure, intravitreal injections, Ozurdex

## Abstract

**Purpose:**

The aim of this study was to estimate the 1‐year costs associated with treating diabetic macular oedema (DME) patients using current intravitreal anti‐vascular endothelial growth factor (anti‐VEGF) biologics compared with the dexamethasone implant.

**Methods:**

We conducted a descriptive cost‐evaluation analysis using data from Oslo University Hospital and literature to compare three different intravitreal drugs for DME: bevacizumab, aflibercept and dexamethasone. Stratification of patients into ‘Naive’ or ‘Switch’ group was based on treatment history. We estimated the costs from healthcare and ‘extended’ healthcare perspectives. Sensitivity analysis evaluated the impact of various parameters.

**Results:**

The average injections per patient per year for the Naive group (bevacizumab), Switch group (aflibercept) and dexamethasone were 9.5, 9.1 and 3.0 respectively. From a healthcare perspective, the 1‐year costs for the Naive group were 15% lower (bevacizumab, €3619), and for the Switch group, 23% higher (aflibercept, €5226) compared with dexamethasone (€4252). The ‘extended’ healthcare perspective showed the cost per patient per year for bevacizumab remained nominally lower in the Naive group, while dexamethasone remained lower for the Switch group (€5116 for dexamethasone, compared to €4987 for bevacizumab and €6537 for aflibercept).

**Conclusions:**

From a primary healthcare perspective, the dexamethasone as a first‐line DME treatment may increase economic costs in settings where bevacizumab is used off‐label. Treating resistant DMEwith dexamethasone may reduce the costs and treatment burden compared with switching to aflibercept.

## Introduction

The global prevalence of diabetes mellitus (DM) is increasing and is projected to affect nearly 700 million people by 2045 (Cho et al. 2018; Saeedi et al. 2019). Following this trend, the number of individuals experiencing DM‐related eye complications, such as diabetic retinopathy (DR), will also likely increase. Diabetic retinopathy (DR) is considered a major cause of vision loss for this patient group (ESC 2019). Adults aged 40–59 years may be among the worst affected groups as these working‐age individuals face sight‐threatening retinopathy (STR) and even blindness. Diabetic retinopathy (DR) progression is also a risk factor for developing diabetic macular oedema (DME), a central macular thickening that causes distortion of the central vision, decreased visual acuity and potentially legal blindness (ESC 2019). The International Diabetes Federation indicates that 20% of Type 1 diabetes (T1D)‐ and 25% of Type 2 diabetes (T2D) patients are at risk of developing DME (Klein et al. 1994; IDF 2020). Sight‐threatening retinopathy (STR) is both avoidable and curable with early detection; however, the complications from DME continue to pose a heavy burden not only on the healthcare system and social services, but also on the scarce monetary and non‐monetary resources.

The complexity of the DME pathophysiology has led to various treatment approaches over the past decades (Lang 2012; Parodi Battaglia et al. 2018). Initially, grid laser photocoagulation was used for DME patients, but this treatment was associated with non‐improved visual acuity, albeit preventing further vision loss (Lang 2012). The greater need for improved visual acuity led to the gold standard treatment in recent years, *that is* the use of anti‐vascular endothelial growth factor (anti‐VEGF) therapies. However, not all patients respond to anti‐VEGFs, such as bevacizumab (Avastin), aflibercept (Eylea) and ranibizumab (Lucentis) (Bressler et al. 2014; Pieramici et al. 2016; Shah & Heier 2016; VanderBeek et al. 2016). Therefore, the persistence of treatment‐resistant‐DME with anti‐VEGFs facilitated the development and introduction of an intravitreal dexamethasone (Ozurdex®; Allergan, Inc., Irvine, CA, USA). The dexamethasone was approved by the United States Food and Drug Administration (FDA), as well as the European Union for DME treatment and its efficacy as monotherapy or in combination with anti‐VEGF has been demonstrated in various studies (Haller et al. 2010; Capone et al. 2014).

Anti‐VEGFs are commonly administered monthly or bimonthly for DME (Boyer & Yoon 2014; DRCRN et al. 2015; Schmidt‐Erfurth et al. 2017). For non‐responsive cases, European guidelines recommend switching drugs after 3–6 injections (Schmidt‐Erfurth et al. 2017). In addition, the dexamethasone is also recommended as an alternative for anti‐VEGF‐resistant patients and has also been suggested as first‐line treatment for DME in pseudophakic eyes, patients with cardiovascular‐related problems and postvitrectomized eyes (Gillies et al. 2014; Fraser‐Bell et al. 2016; Schmidt‐Erfurth et al. 2017). Compared with anti‐VEGFs, the dexamethasone is administered less frequently, sometimes requiring only two injections over 12 months; however, studies have shown better outcomes with reinjection intervals of <5 months, *that is* an average of three injections per year (Haller et al. 2011; Mastropasqua et al. 2015; Sarao et al. 2017; Urbančič & Topčić 2019). Dexamethasone nevertheless harbours a risk of adverse events, such as increased intraocular pressure and cataract formation in phakic eyes (Boyer & Yoon 2014; Gillies et al. 2014; Fraser‐Bell et al. 2016).

Several studies have indicated that different anti‐VEGFs, for example bevacizumab, aflibercept and ranibizumab, achieve comparable effect and safety profiles (DRCR et al. 2015; Heier et al. 2016). Furthermore, evidence show that strict 3‐ to 5‐month reinjection intervals of the dexamethasone have the potential to sustain fewer and more manageable adverse events while achieving a similar therapeutic effect as the anti‐VEGFs (Mastropasqua et al. 2015; Matonti et al. 2016; Callanan et al. 2017; Sarao et al. 2017; Urbančič & Topčić 2019; Rosenblatt et al. 2020). Given the increasing evidence of a similar drug effect and the use of multiple alternative drugs for DME, an economic assessment is warranted to help estimate the economic implications of these DME treatment alternatives. In this study, we aimed to estimate the 1‐year costs associated with treating DME patients using either current treatment regimens involving anti‐VEGF therapies (bevacizumab and aflibercept) compared with the dexamethasone for two sub‐groups of pseudophakic DME patients: Naive patients initiating treatment with bevacizumab and Switch patients converting from bevacizumab to aflibercept.

## Methods

Our study involved retrospective, single‐centre quality registry data from Oslo University Hospital (OUH), Norway, combined with published literature, national fee schedules and listed drug prices to compare the 1‐year economic costs of three alternative DME treatments. The use of registry data was approved by the institutional data protection officer in accordance with the General Data Protection Regulation. Data from all intravitreal injections for the years 2017 and 2018 were collected. Although bevacizumab is the first line of treatment for centre‐involving DME and baseline visual acuity above 20/50 and aflibercept is the first line of treatment for patients with visual acuity below 20/50, we included only first‐line treatment (*i.e*. Naive group) assuming bevacizumab. Patients who failed to respond after 4–6 consecutive monthly injections of bevacizumab are switched (*i.e*. Switch group) to another anti‐VEGF, usually aflibercept. We assume the dexamethasone could be provided to either group of patients or used data from published literature to quantify expected resource use in the first‐year. We identified, quantified and valued the costs from a healthcare perspective (*i.e*. direct medical costs) and an ‘extended’ healthcare perspective (*i.e*. direct medical and direct non‐medical costs including patient time and transportation), as recommended by the National Medicines Agency (NoMA 2020).

### Study design and analytic sample

Based on assumptions of similar drug effectiveness (DRCR et al. 2015; Heier et al. 2016), we employed a cost‐minimization approach to estimate the comprehensive 1‐year costs associated with treating DME patients using current treatment regimens involving anti‐VEGFs for Naïve (bevacizumab) or Switch patients (aflibercept) compared with a hypothetical scenario of introducing dexamethasone as a treatment alternative for the two different patient groups. A cost‐minimization study is considered appropriate where two or more comparative interventions or drugs have similar outcomes (Brown et al. 2003; Drummond et al. 2005). Patients were stratified into (1) a Naive group, *that is* unilateral treatment initiated by bevacizumab and completed 1 year of treatment and (2) a Switch group, *that is* patients who switched from bevacizumab to aflibercept and remained on aflibercept for at least 1 year. We included all costs that were associated with each group within a 1‐year time horizon, starting from the time of treatment decision—that is either initiating treatment or switching treatments.

### Data collection and follow‐up

**Fig. 1:**
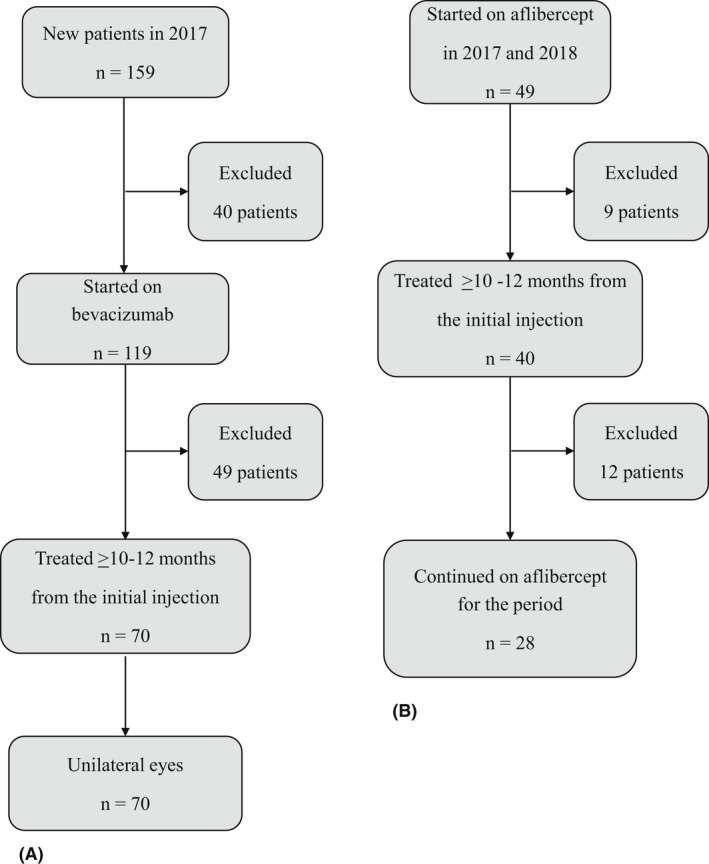
Study design for the Naïve (A) and Switch patients (B).

Anonymized data for the period for 2017 and 2018 were collected using a generated index as patient identity to calculate one patient year cost following treatment initiation or switching. The analysis included only patients receiving unilateral eye treatment. Naive patients included those who were initiated on bevacizumab in 2017 to ascertain a 1‐year cost. Patient lacking data up to 1 year (minimum 10 months) from the first injection were excluded. The drugs used intermittently, such as ranibizumab or aflibercept, were also recorded. Switch patients' group included only those treated prior to 2017 with any other drug except aflibercept and had switched to aflibercept in 2017. Only those who continued treatment for the 1‐year period were included in the analysis (Fig. 1). The mode of treatment/drug used before or after any visit if not registered was assumed for adjustment of missing data. None of the included patients had more than one visit of missing drug information. The quality register data did not contain information on whether the patients were phakic or pseudophakic; therefore, we assumed that the patients included in the study were pseudophakic, in order to compare the suggested first‐line treatments for these patients, or dexamethasone as a switch alternative.

As relatively few patients are actually treated with dexamethasone at OUH, we used data from the literature on injection frequency to estimate the expected resource use and cost of dexamethasone (Mastropasqua et al. 2015; Sarao et al. 2018; Urbančič & Topčić 2019). In line with the literature, we assumed three dexamethasone injections were given over the 1‐year period, irrespective of Naive or Switch patient groups. In addition, we assumed that every injection of dexamethasone was followed by one visit to measure intraocular pressure and that 22% required topical pressure‐lowering treatment (Zarranz‐Ventura et al. 2020). Optical coherence tomography (OCT)/visual acuity measures were assumed to take place on the same day as the injection for all drugs.

### Cost data

The economic costs of each treatment regimen in the primary healthcare perspective included drug cost, injection visits and the follow‐up visits. For patients receiving the dexamethasone implants, these costs additionally included the costs for intraocular pressure management or pressure reducing eye drops. The cost of the diagnostic tests was assumed to be independent of the selected treatment and was not included.

We used unit cost data as suggested by the Norwegian Medicines Agency (NoMA) for drug reimbursements (Table 1) (NoMA 2018a, 2018b). All unit costs were converted to Euros as per 16 December 2020 exchange rate, *that is* 10.58 Norwegian Kroner (NOK) equal to 1 EUR. Published drug list prices, after removing value‐added tax (VAT), were used to calculate the cost of dexamethasone, bevacizumab and aflibercept. For all current anti‐VEGF biologics, ready to use syringes are compounded from vials at the OUH hospital pharmacy (Sivertsen et al. 2018; Lode et al. 2019); subsequently, the drug costs are calculated based on using each bevacizumab vial for 40 injections and each aflibercept vial for 2.5 injections. The drug cost also included a compounding cost estimated to €30 per drug syringe. The dexamethasone is individually packed and was not adjusted for the vial cost; however, intraocular pressure management (examination and topical medications) cost was added equivalent to the number of dexamethasone visits. The cost of cataract surgery has been assumed to be the product of the DRG weight and its value (Helsedirektoratet 2020).

**Table 1:** Drug costs and unit costs.

Cost item	Unit cost (excluding VAT)	Reference
Bevacizumab: vial, 4 ml, 25 mg/ml	€ 36.30	NoMA (2018a)
Aflibercept: vial, 0.1 ml, 40 mg/ml	€ 310.10	NoMA (2018a)
Dexamethasone: 700 μg implant	€ 1020.21	NoMA (2018a)
Hospital Pharmacy compounding cost per syringe	€ 30	Estimate from Oslo University Hospital
Cost of injection	€ 130.43	NoMA Unit cost database (2018b)
Visit to specialist	€ 66.64	NoMA Unit cost database (2018b)
Visit to specialist nurse	€ 46.88	NoMA Unit cost database. Cost per hour (2018b)
Transport cost per journey	€ 55.20	NoMA Unit cost database (2018b)
Patient clinic time	€ 22.40	NoMA Unit cost database. Cost per hour (2018b)
Cataract surgery	€ 896.24	DRG39Q (Helsedirektoratet 2020)

16 December 2020, conversion rate 10.58NOK per 1€.

We further expanded our analytical perspective as recommended by the national guidelines for healthcare intervention evaluations to include transport cost for the journey to and from the healthcare centre; therefore, the transport cost was multiplied by 2 to cover the round trip. While the patient's time at the facility and the patient's time required for the roundtrip journey to/from the eye facility was averaged at 90 min, the patient's time cost was multiplied by 1.5 for both patient groups (Naive and Switch). The product was multiplied by the number of visits in each group and added to the total cost of the respective group. We applied the same to dexamethasone visits and added the intraocular pressure visits multiplied by the time cost and transport cost.

### Analysis and outcomes

We calculated the expected 1‐year treatment cost for each patient group–treatment combination by estimating the number of visits for either of the drugs used. This aided in calculating average visit cost or injection cost and the post‐injection care for the dexamethasone group. We thereafter adjusted the drug cost for the vial compounding; then, the product was added to injection cost and associated follow‐up cost for the 1‐year period. Based on the register data for bevacizumab and aflibercept, calculations were undertaken using the same approach to calculate the 1‐year cost per patient for dexamethasone. The primary outcome included the 1‐year costs per patient per year, as well as descriptive outcomes such as the number of patients in the Naive and Switch group, the mean number of injections for the different drugs per year and the mean duration between each individual drug injections.

We stratified costs as drug cost and resource utilization cost in order to calculate the difference between the drugs and the total cost difference per patient per year. Resource utilization included visit cost to the specialist, injection cost, OCT cost and intraocular pressure management cost in case of dexamethasone. The patient transport cost and the calculated patient time cost were added to determine the ‘extended’ healthcare perspective cost. We aggregated the total to estimate the overall cost per patient per year from two perspectives for each drug in the ‘extended’ healthcare perspective and the healthcare perspective, and we differentiated each stratum between the drugs either bevacizumab (Naive) or aflibercept (Switch) total cost from dexamethasone total cost in establishing the less expensive treatment. The analysis was performed using Microsoft Excel in Office365.

### Sensitivity analysis

To ascertain the robustness of our analysis, we included a number of one‐ and two‐way sensitivity analyses (Drummond et al. 2005; Halpern & Pandharipande 2017). First, even though a strict adherence is recommended for dexamethasone treatment, we explored the impact of assuming a range of yearly injections (*i.e*. 2.5–4) in both Naive and Switch groups (Mastropasqua et al. 2015). In addition, the variability in the number of injections could also inform the follow‐up years as monitoring DME patients exceeds the first year of treatment. Second, in the two‐way sensitivity analysis, we evaluated the uncertainty in delivering anti‐VEGFs monthly, bimonthly or quarterly in comparison with a range of delivering dexamethasone injections between 2.5 and 4 times per year. The bounds explored in the sensitivity analysis were informed by a supplementary analysis that estimated the average number of injections for a subset of data that followed a cohort over two consecutive years (See Supplementary Appendix).

In the treatment setting, intraocular pressure management could be administered by either ophthalmologist or a specialist nurse. Therefore, we performed a two‐way sensitivity analysis estimating this scenario on the number of dexamethasone injections per year in each group. As drug costs differ, we conducted a two‐way sensitivity on the drug costs in the Naive group by assuming a reduction or an increase in the cost by 20% and also included a scenario of reducing the cost by 40% (Ross et al. 2016; Parikh et al. 2019). We additionally estimated the impact of reducing or increasing the injection cost and the other unit cost by 50%. Lastly, we included a treatment regimen possibility which may result in treating phakic patients. In this sensitivity analysis, we included the probability of developing cataract cost in the aggregated cost. This was informed by the MEAD and BEVORDEX studies; however, only the BEVORDEX‐study probabilities were used, as the treatment regimen met the study assumed treatment effects (Boyer & Yoon 2014; Gillies et al. 2014; Mastropasqua et al. 2015; Matonti et al. 2016; Callanan et al. 2017; Sarao et al. 2017; Urbančič & Topčić 2019).

## Results

### Current treatment use by patient group

In 2017, 159 patients at OUH were identified as Naive patients and 49 as Switch patients. In the Naive group, 119 patients initiated bevacizumab (40 patients on another anti‐VEGF) and 70 (59%) met the inclusion criteria for this study; 49 (41%) did not continue with the treatment (with a mean (SD) of 2.5 (±2.1) injections before treatment termination). Half of the included patients continued on bevacizumab for the 1‐year period, while 35 of the patients received other drugs intermittently (*i.e*. aflibercept or ranibizumab) (Table 2). In total, there were 665 injections for the 70 patients, or 70 eyes included in the Naive group during the study year with an average of 9.5 (±3.1) injections/visits. Bevacizumab patients who continued with the drug received on average 8.3 (±3.3) injections per year, while those registered with other drugs intermittently had 11 (±2.4) injections in that year, having received at least 4.7 (±2.7) injections before the use of any other drug. The interval between the drugs varied, as those patients' receiving bevacizumab had on average 44 days in between injections, while those receiving aflibercept had on average 37 days in between injections.

**Table 2:** Current treatment modalities for diabetic macula oedema with the number of patients treated at the Oslo University Hospital in the Naive group (initiated on bevacizumab) and the Switch group (switched to aflibercept) in 2017 and the number of injections during the 1‐year period.

Patient group			Number of patients		Average number of injections for 1 year (± standard deviation)
Naive	Initiated on bevacizumab in 2017	All drug treatments	70		9.5 (±3.1) during the 1‐year period (on bevacizumab, aflibercept or ranibizumab)
		Continued on bevacizumab for 1 year		35 (50%)	8.3 (±3.3) during the 1‐year period (on bevacizumab)
		Other registered drugs		34 (48.6%) aflibercept	4.7 (±2.8) on bevacizumab, 6.3 (±3.1) on aflibercept or ranibizumab
				1 (1.4%) ranibizumab	
Switch	Switched to aflibercept in 2017	Continued on aflibercept for 1 year after switch	28		9.1 (±2.8). during the 1 year after switch (to aflibercept)

Naive group: patients initiated on bevacizumab at the start of the inclusion period in 2017; Switch group: patients switched to another anti‐VEGF than bevacizumab, usually aflibercept.

In the Switch group, 40 patients (81.6%), or 40 eyes, completed the 1‐year inclusion period from the 49 patients. However, during the study year, one‐quarter of the patients switched back to bevacizumab or ranibizumab and were, therefore, excluded. The patients (n = 28) who remained on aflibercept for the one‐year study period were included in the analysis and had received 9.1 (±2.8) injections on average during the study period (Table 2 and Fig. 1). Injection interval for these patients was on average 43 days (±32.5).

### One‐year economic costs of treatment for DME patients

For first‐line Naive patients from a healthcare perspective, the use of dexamethasone yielded a higher 1‐year expected healthcare expenditure per patient than patients initiating bevacizumab (€4252 for dexamethasone *vs*. €3619 for bevacizumab) (Table 3). Approximately 72% of these economic costs in the dexamethasone group were due to drug cost (€3061), while in the bevacizumab group, 68.6% of the economic costs were attributed to resource utilization due to the higher frequency of visits and injections required by a bevacizumab regimen. From the ‘extended’ healthcare perspective, the overall average cost per patient per year of dexamethasone was found to be approximately similar to almost 3.5 bevacizumab drug cost. Dexamethasone provided cost savings compared with bevacizumab for non‐drug‐related costs such as resource use, transportation and time spent receiving treatments.

**Table 3:** One‐year baseline cost per patient eye to treat patients with diabetic macula oedema using either standard of care in Naive patients (bevacizumab or dexamethasone (DEX)) or Switch patients (aflibercept or dexamethasone (DEX)).

	DEX	Naive patient group		Switch patient group	
		Bevacizumab	DEX—cost difference	Aflibercept	DEX—cost difference
Drug cost	€3061	€1114	€1946	€2824	€237
Resource utilization	€1191	€2505	−€1314	€2402	−€1211
Transport cost	€662	€1048	−€386	€1005	−€343
Patient time	€202	€319	−€118	€306	−€104
Total cost per year per patient (healthcare perspective)	€4252	€3619	€632	€5226	€974
Total cost per year per patient (‘extended’ health care perspective)	€5116	€4987	€128	€6537	−€1421

For the Switch patients, the dexamethasone treatment had an average drug cost of €3061 per patient that was 7.7% higher than that for aflibercept (€2824); however, these drug costs were more than offset due to the cost‐saving dexamethasone provided on resource utilization (Table 3). Subsequently, the total cost per patient per year of treating Switch patients with dexamethasone was 18.6% lower compared with aflibercept, yielding €974 in cost saving per patient per year. The benefits were even higher under the ‘extended’ healthcare perspective for dexamethasone, as the cost savings for transportation and patient time increased the total cost savings to €1421.

### Sensitivity analyses

**Fig. 2:**
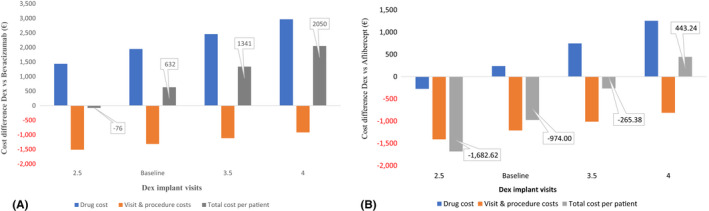
One‐way sensitivity analysis for the 1‐year cost differences per patient for Dexamethasone implant (DEX) compared with Bevacizumab by the number of DEX visits for Naive patients (A) and for DEX compared with Aflibercept by number of DEX visits for Switch patients (B) from a healthcare perspective. [Colour figure can be viewed at wileyonlinelibrary.com]

From a healthcare perspective, when we explored the impact of lower and upper bounds of the number of dexamethasone injections (*i.e*. between 2.5 and 4), we found that any fewer than or equal to 2.5 dexamethasone visits per year yielded lower total cost per patient per year among Naive patients compared with bevacizumab (Fig. 2A). In contrast, for the Switch group, dexamethasone remained cost saving until we increased the number of dexamethasone visits to 4 per year (Fig. 2B).

**Fig. 3:**
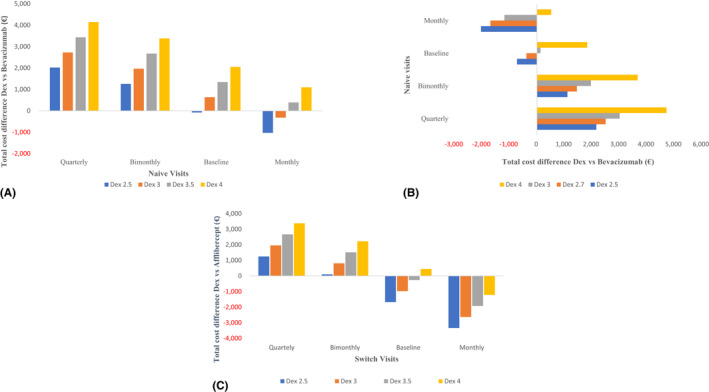
Two‐way sensitivity analysis for the total 1‐year cost differences per patient for Dexamethasone implant (DEX) compared with Bevacizumab by the number of visits for both drugs in Naive patients in the primary healthcare perspective (A) and ‘extended’ healthcare perspective (B), as well as on Aflibercept compared with DEX by the number of visits in Switch patients in a primary healthcare perspective (C). [Colour figure can be viewed at wileyonlinelibrary.com]

In a two‐way sensitivity analysis among Naive patients, bevacizumab remained less costly if visits were less frequent than baseline assumptions, *that is* bimonthly and quarterly. Meanwhile, the total average cost per year was lower for dexamethasone when we assumed fewer, *that is* 2.5, dexamethasone visits per year compared with monthly bevacizumab (Fig. 3A). From an ‘extended’ healthcare perspective assuming higher than baseline bevacizumab injections, the average cost per patient per year became cost saving with <4 dexamethasone per patient per year. When we assumed fewer than baseline bevacizumab injections, the total average cost per patient per year was higher regardless of the number of dexamethasone injections per year (Fig. 3B). When we used our exploratory analysis (see Supplementary Appendix) to inform bevacizumab injection frequency over a 2 consecutive‐year period, *that is* 16.4 injections, the total average costs over the 2‐year period remained higher for dexamethasone (assuming 6 total dexamethasone injections over the 2‐year period). However, if the number of dexamethasone injections also decreased in year 2, the dexamethasone strategy becomes cost saving compared with receiving bevacizumab (Figure S1 and S2).

In a two‐way sensitivity analysis among Switch patients, we found that when aflibercept was administered less frequently, either bimonthly or quarterly, the total average cost per patient per year was higher in all scenarios of dexamethasone injections delivered per year. In contrast, dexamethasone was less costly in circumstances where aflibercept was administered monthly, irrespective of the number of dexamethasone injections provided per year, while holding all other parameters constant (Fig. 3C).

**Fig. 4:**
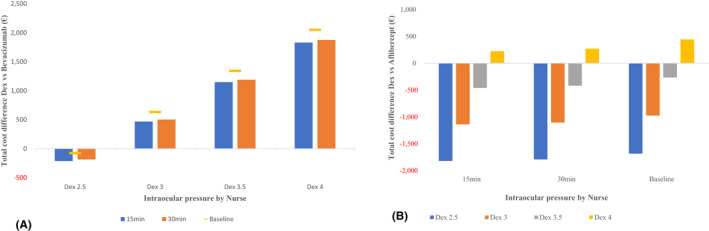
Two‐way sensitivity analysis for the total 1‐year cost difference per patient for Dexamethasone implant (DEX) compared with Bevacizumab when intraocular pressure measurement is performed by an eye specialist for the Naive patients (A) and DEX compared with Aflibercept when the intraocular pressure measurement is performed by an eye specialist for the Switch patients (B) in a primary healthcare perspective. [Colour figure can be viewed at wileyonlinelibrary.com]

Dexamethasone costs remain lower if the intraocular pressure measurement was performed by a nurse with the 2.5 injections per year, despite time taken by the nurse to offer this service (15 or 30 min). With 3 or more dexamethasone injections per year, the cost savings were moderated, yet still remained lower than the use of the eye specialist in Naive patients (Fig. 4A). Among the Switch patients, there is a cost saving incurred when using a nurse to provide the intraocular pressure measurement for dexamethasone patients, except when dexamethasone visits were assumed to be 4 or more per year. Our finding remains robust when we assumed the nurse provided the intraocular pressure management in 15 or 30 min (Fig. 4(B)).

**Fig. 5:**
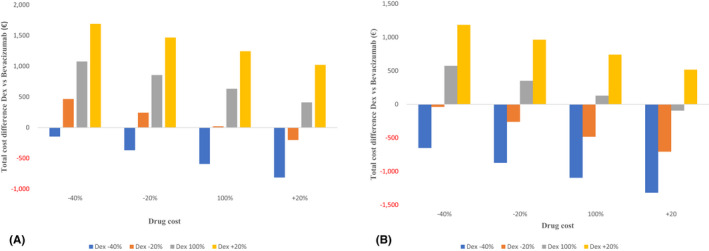
Two‐way sensitivity analysis for the total 1‐year cost difference per patient of Dexamethasone implant (DEX) compared with Bevacizumab by the drug cost in Naive patients. Primary‐ (A) and extended‐ (B) healthcare perspective. [Colour figure can be viewed at wileyonlinelibrary.com]

When we explored the impact of varying the drug costs in the Naive group, we found that a 20% decrease in the dexamethasone drug cost would lower the total cost difference in the event of an increase of 20% of bevacizumab. However, as the drug cost dropped by 40%, there was lower total cost per patient irrespective of the cost changes among the bevacizumab patients (Fig. 5A). Accordingly, in the ‘extended’ healthcare perspective, we found similar trends when we reduced dexamethasone drug cost either by 20% or 40% (Fig. 5B).

When we decreased the visit and injection unit costs in the two groups by 50%, dexamethasone remained more costly for Naïve patients and cost saving for Switch patients (Table 4). We found similar trends when we increased the visit and injection unit costs in the two groups. Finally, when we included an assumption of cataract development costs for 6% of the patients in the Switch group, we found a higher cost per patient in the dexamethasone group; however, dexamethasone remained the less costly treatment. The results remained similar when we assumed up to 3.5 or 4 dexamethasone injections per year and assumed either baseline or monthly injections rates with aflibercept. In contrast, when we considered less frequent bimonthly or quarterly aflibercept injections, dexamethasone cost exceeded the costs of aflibercept in the ‘extended’ healthcare perspective (results not shown).

**Table 4:** Sensitivity analyses evaluating the impact of the injection or treatment visits costs on total costs for bevacizumab or aflibercept compared with dexamethasone implant (DEX) in Naive or Switch patients from a healthcare perspective.

Visit and injection	Naive patients			Switch patients		
	DEX (€)	Bevacizumab (€)	Difference (€)	DEX (€)	Aflibercept (€)	Difference (€)
Unit costs: −50%	3856	2683	1173	3856	4328	−472
Baseline	4252	3619	632	4252	5226	−974
Unit costs: +50%	4647	4555	92	4647	6123	−1476

## Discussion

Our study estimated 1 year cost of treating Naive patients initiated on bevacizumab compared with dexamethasone and non‐responding Switch patients with aflibercept compared with dexamethasone. Assuming a healthcare perspective for Naive patients, we found higher overall costs per patient per year for dexamethasone (€4252) compared with patients initiating bevacizumab (€3619); however, when including costs related to patient transportation and time to undergo treatments, we found only nominal cost differences (€5116 dexamethasone *vs*. €4987 bevacizumab). The high cost is attributed mainly with the dexamethasone drug cost, which is over twice the cost of resource utilization (€3061 drug cost and €1191 resource utilization cost). For non‐responding Switch patients, we found that 1‐year costs for dexamethasone provided cost saving of €974 compared with aflibercept (€1421 from an ‘extended’ healthcare perspective).

Our findings were comparable to a study performed in an Italian healthcare institution, although the treatment frequency considered in that study was lower compared with our real‐world hospital utilization data (Foglia et al. 2018). To the best of our knowledge, this is the first study to evaluate the 1‐year healthcare expenditures for DME patients that are either Naive or Switch patients involving the drugs compared. Other studies have evaluated either cost‐effectiveness of anti‐VEGFs without dexamethasone or budget impact analysis with different scenarios (Régnier et al. 2015; Kourlaba et al. 2016; Ross et al. 2016; Foglia et al. 2018).

In comparison with the anti‐VEGF therapy, dexamethasone may lower the burden for patient visits and the injection frequency. However, dexamethasone may cause high intraocular pressure with almost 22% of the patients exhibiting elevated pressure requiring medical treatment, as well as cataract progression. These adverse events may necessitate more frequent care and close monitoring among high‐risk patients (Gillies et al. 2014; Shah & Heier 2016; He et al. 2018; Zarranz‐Ventura et al. 2020). Our sensitivity analysis found that at our baseline bevacizumab frequency of care or lower, beyond 3 dexamethasone visits increased the cost of annual treatment on average in both patient groups. Nevertheless, this cost may be moderated by utilizing a nurse to manage the intraocular pressure measurement in patients. Importantly, among the Switch patients, our results on intraocular pressure management indicate that dexamethasone yields higher cost saving for <4 dexamethasone visits or injections per year. The frequency of injections has been associated with various other adverse events including endophthalmitis or lens damage. Even though these are extremely rare, an inefficient preparation or inexperienced staff involved in the delivery of the treatment may lead to higher costs (Meyer et al. 2010). Given the very low incidence of such events, we excluded them from the calculations.

The effect of injection and visit costs was estimated in a two‐way sensitivity analysis; we found that a 50% reduction in resource use showed no change in the baseline results in the two groups studied. However, we found there were similar costs when we assumed a 50% increase in the number of visits and injections for both drugs among the Naive patients. Meanwhile, in the Switch group, dexamethasone remained cost saving in all scenarios evaluated despite variations in aflibercept injection cost and visit assumptions. Considering that monitoring of DME patients extends beyond 2 years, this will then increase the resource use considerably. Even though on exploring, our subset data indicated fewer injections in subsequent years (Boyer & Yoon 2014; Campbell et al. 2014; Parikh et al. 2019; Billioti de Gage et al. 2021) but higher average costs, suggesting for prolonged care, dexamethasone would be favourable in costs. The initial year being less costly for Naïve patients initiating on bevacizumab, clinicians could consider switching patients under monitoring beyond one year to dexamethasone to save on costs.

A previous study estimating the cost‐effectiveness of anti‐VEGFs indicated that some of the drug prices would need to fall by over 60% to be considered cost‐effective compared with bevacizumab in accordance with the US recommended threshold (Ross et al. 2016). However, another study showed that the prices for some identified ophthalmic drugs have been declining in other developed countries, yet the cost is still considered high to reach a level of reduced cost burden (Parikh et al. 2019). Our sensitivity analysis of 20% decrease in dexamethasone drug prices found that Naive patients' economic costs yield similar cost at baseline or cost saving with higher bevacizumab drug costs. In addition, with reduction in about 40% in dexamethasone costs, the economic cost benefit increases regardless of the price changes in bevacizumab. Nevertheless, the bevacizumab benefit on the practice of compounding the drugs that significantly reduce its cost in the public health care systems (Sodré et al. 2019). In our study, the Naive group was evaluated as patients initiating DME treatment with bevacizumab. However, as in most countries, bevacizumab has not been approved for treatment of DME and is used off‐label; therefore, other anti‐VEGF drugs such as ranibizumab or aflibercept are often used to initiate treatment for this patient group (Foglia et al. 2018). In such scenarios and assuming the cost of ranibizumab is higher than bevacizumab and treatment effects are relatively similar between drugs, dexamethasone may likely be cost saving in the first year (especially from an ‘extended’ healthcare perspective), due to the reduced number of visits and the drug cost comparatively.

As stated in several studies, the increased benefits to the DME patients have been the result of the multimodal approach in their treatment. However, there are existing differences on how and when to switch between treatments modalities. Optimal outcomes have been documented for early switching (*i.e*. before 6 months) for anti‐VEGF‐resistant patients (Schmidt‐Erfurth et al. 2017; Demir et al. 2020). In the Naive group, which included 50% patients that switched after 4.7 bevacizumab injections on average, the group maintained a lower cost compared with dexamethasone‐only treatment in the primary healthcare perspective while, from an ‘extended’ healthcare perspective, the average costs were similar with 3.5 bevacizumab drug cost. Nevertheless, as seen in the Switch group, the continued treatment, which includes dexamethasone, may overall provide savings to the healthcare system. Therefore, for patients that are likely to require frequent prolonged DME monitoring, dexamethasone could be a preferred choice after the initial year.

Even though this study assumed patients included to be pseudophakic, treating phakic patients may increase the cost due to a higher probability of developing cataract with the use of dexamethasone. In the BEVORDEX study, 6% of the patients in the first 12 months were treated for cataracts among the phakic eyes receiving dexamethasone (Gillies et al. 2014). These adverse events can impose even higher costs for Naive patients if treated with dexamethasone as a first‐line therapy. When we included the cataract surgery cost among the Switch patients in a sensitivity analysis, we did not find an important impact in the cost for dexamethasone. Importantly, this finding is robust when the dexamethasone treatment is administered up to 4 times per year as compared to our baseline assumption for aflibercept (*i.e*. 9 injections per year) or even when we assumed monthly aflibercept injections. However, if patients continue on a medication over many years, which is likely for DME patients, the risk of cataract may increase beyond what we assumed. In addition, due to the interaction of DM as a risk factor for developing cataract, DME patients may still experience cataract development irrespective of the treatment drug used. (Boyer & Yoon 2014; Fraser‐Bell et al. 2016).

There is also an extended indirect burden on healthcare utilization that could be due to the type of drug used—for instance, the data from the OUH indicate an interval between 37 and 44 days between injections in both Naive and Switch patients. Other studies have shown the interval between injection for dexamethasone to be up to 145 days on average (Mehta et al. 2017; Rosenblatt et al. 2020). As hospital demands for injections have increased with time (Jørstad et al. 2019), the length of time between each dexamethasone reinjection provides an opportunity to increase capacity to treat new patients by almost threefold. In general, healthcare institutions should be prepared to be able to provide access to increased patient treatment and identify treatments that reduce healthcare expenditures.

Our study had a higher average number of injections or visits than other clinical practice studies (Campbell et al. 2014; Patrao et al. 2016) even though we considered only one eye for all patients irrespective of the bilateral injection in almost 30% of the patients visits. The cost of treating unilateral or bilateral eye in a visit brings equal reimbursement for the procedure at the OUH. The higher average of the visits could be due to the universal coverage for the Norwegian healthcare system. The system allows easy access to medical care though with a small co‐payment share with a price cap that is low enough to accommodate patient needs. Therefore, we assume that the effects are close to those of the clinical trials outcome strengthening our results on the assumptions included (Nguyen et al. 2012; Korobelnik et al. 2014; Wells et al. 2016).

There are several limitations of the current study. One limitation is that all data are not from the same study setting and relied on some published data as estimates. For example, we relied on the literature to inform the expected annual number of injections for dexamethasone. However, we explored plausible ranges in our base case assumptions. In addition, the majority of our referenced studies that carried out randomized clinical trials in different settings found similar outcomes (Mehta et al. 2017; Rosenblatt et al. 2020). In addition, we also assumed that our patient population was pseudophakic. As the intensity of the treatment regimen or drug choice does not vary for phakic or pseudophakic patients, we do not expect this assumption to bias our results. Moreover, as studies have already indicated the initiation of dexamethasone in pseudopakic patients, calculating this cost comparatively as initiated on bevacizumab was probably informative in the decision making (Gillies et al. 2014; Fraser‐Bell et al. 2016; He et al. 2018). Nevertheless, we performed a sensitivity analysis under the assumption of including phakic patients to account for the possible adverse events, which did not impact our conclusions as long as aflibercept injection interval was bimonthly or more frequent.

We did not include some direct non‐medical costs such as those associated with family or friends accompanying patients to and from appointments. Including spillover effect costs would increase the total costs associated with all treatment modalities but could results in narrowing the incremental costs between dexamethasone and bevacizumab due to the lower frequency of visits in the treatment with dexamethasone compared with bevacizumab. Although these data were not available, expert consultations with the OUH ophthalmic department found that the proportion of patients being accompanied by the clinic was small (NoMA 2020). Including these additional costs would have no impact on costs estimated from the primary healthcare perspective. In addition, we considered lower visual acuity that could occur during treatment—in such a case, further indirect costs may be relevant to include. In our study, considering the groups included, an indirect cost such as for vision loss is highly unlikely.

We also acknowledge that evaluation studies should include a probabilistic analysis to quantify credible intervals; however, in this cost‐minimization study, the uncertainties in the cost parameters could be broadly explored through one‐ and multi‐way deterministic analyses to provide the impact of parameters on the outcomes (Halpern & Pandharipande 2017).

## Conclusions

Treating DME patients poses burden to healthcare systems and can be expected to increase as the burden of DM continues to increase. Our study indicates that the economic costs of intravitreal injections vary by patient group and treatment modality. The treatment practice of dexamethasone for pseudophakic patients as first‐line treatment for DME may increase economic costs in settings where bevacizumab is used off‐label but is comparable to the average cost for bevacizumab from an ‘extended’ healthcare perspective, as a dexamethasone treatment protocol requires fewer injections, hospital resources and patient time compared with anti‐VEGFs. For non‐responding Switch patients, dexamethasone is likely to yield lower annual costs in comparison with aflibercept and may enable increasing capacity to meet the growing need of intravitreal injections.

16 December 2020, conversion rate 10.58NOK per €.

Naive group: patients initiated on bevacizumab at the start of the inclusion period in 2017; Switch group: patients switched to another anti‐VEGF than bevacizumab, usually aflibercept.

## Supporting information


**Figure S1.** Two‐way sensitivity analysis for the total two‐year cost differences per patient for dexamethasone (DEX) compared with bevacizumab by the number of visits for both drugs in Naive patients in the ‘extended’ healthcare perspective.

**Figure S2.** One‐way sensitivity analysis for the two‐year cost differences per patient for dexamethasone (DEX) compared with bevacizumab by the number of dexamethasone visits for Naive patients in the ‘extended’ healthcare perspective.

**Table S1.** Treatment modalities and cost for diabetic macula oedema with the number of patients treated at the Oslo University Hospital in the Naive group (initiated on bevacizumab in 2016–2018).
