# Supplementary figures and images for: Healthcare expenditure of intravitreal anti‐vascular endothelial growth factor inhibitors compared with dexamethasone implant for diabetic macular oedema
Source: Acta Ophthalmol. 2022 Apr 25;100(8):e1630–40. doi: 10.1111/aos.15151 (PMC9790387; doi:10.1111/aos.15151)

Figure S1

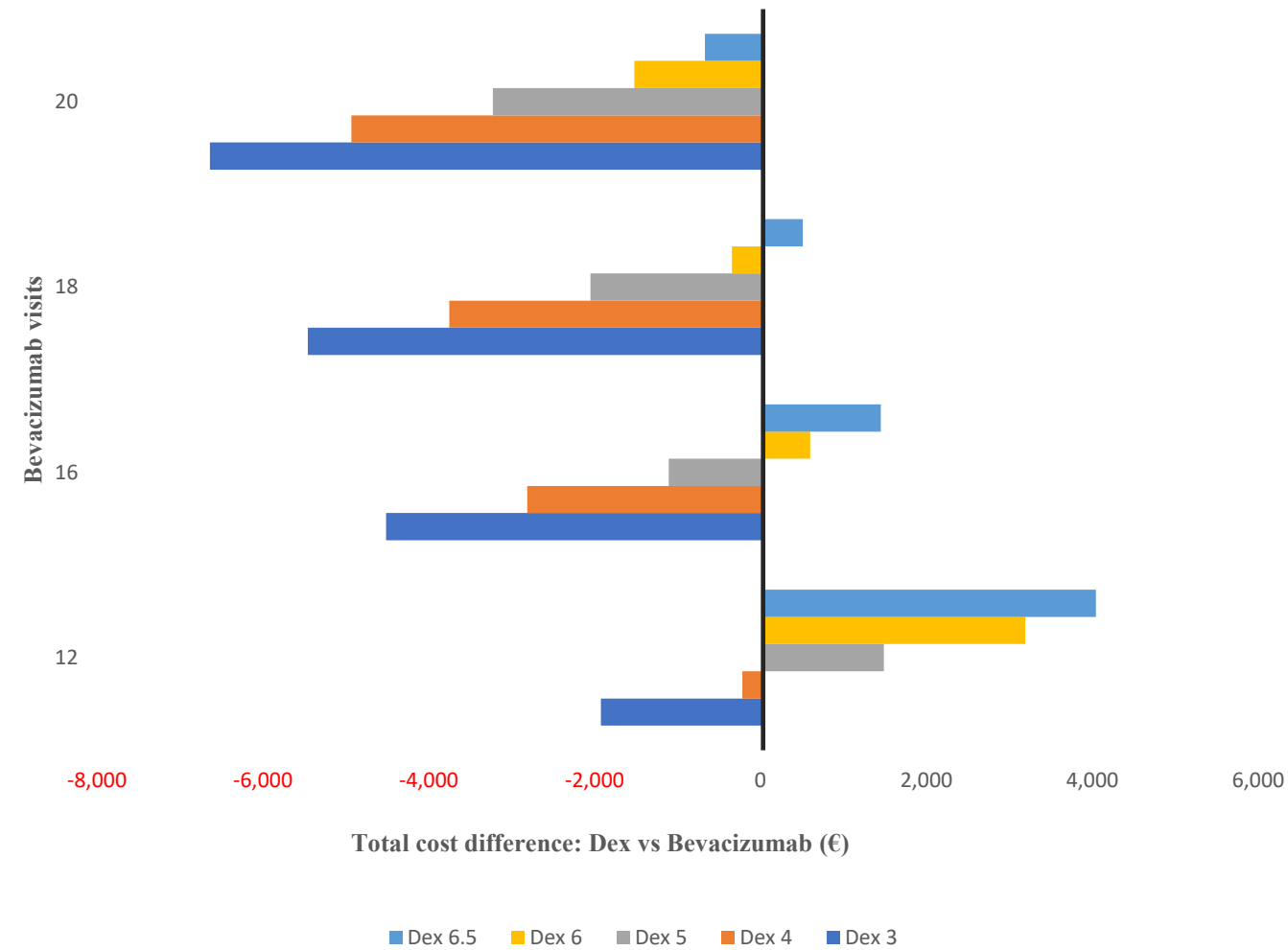

Supplement: Supplementary file 1 — Figure S1. Two‐way sensitivity analysis for the total two‐year cost differences per patient for dexamethasone (DEX) compared with bevacizumab by the number of visits for both drugs in Naive patients in the ‘extended’ healthcare perspective. [file AOS-100-e1630-s002.pdf]

Figure S2

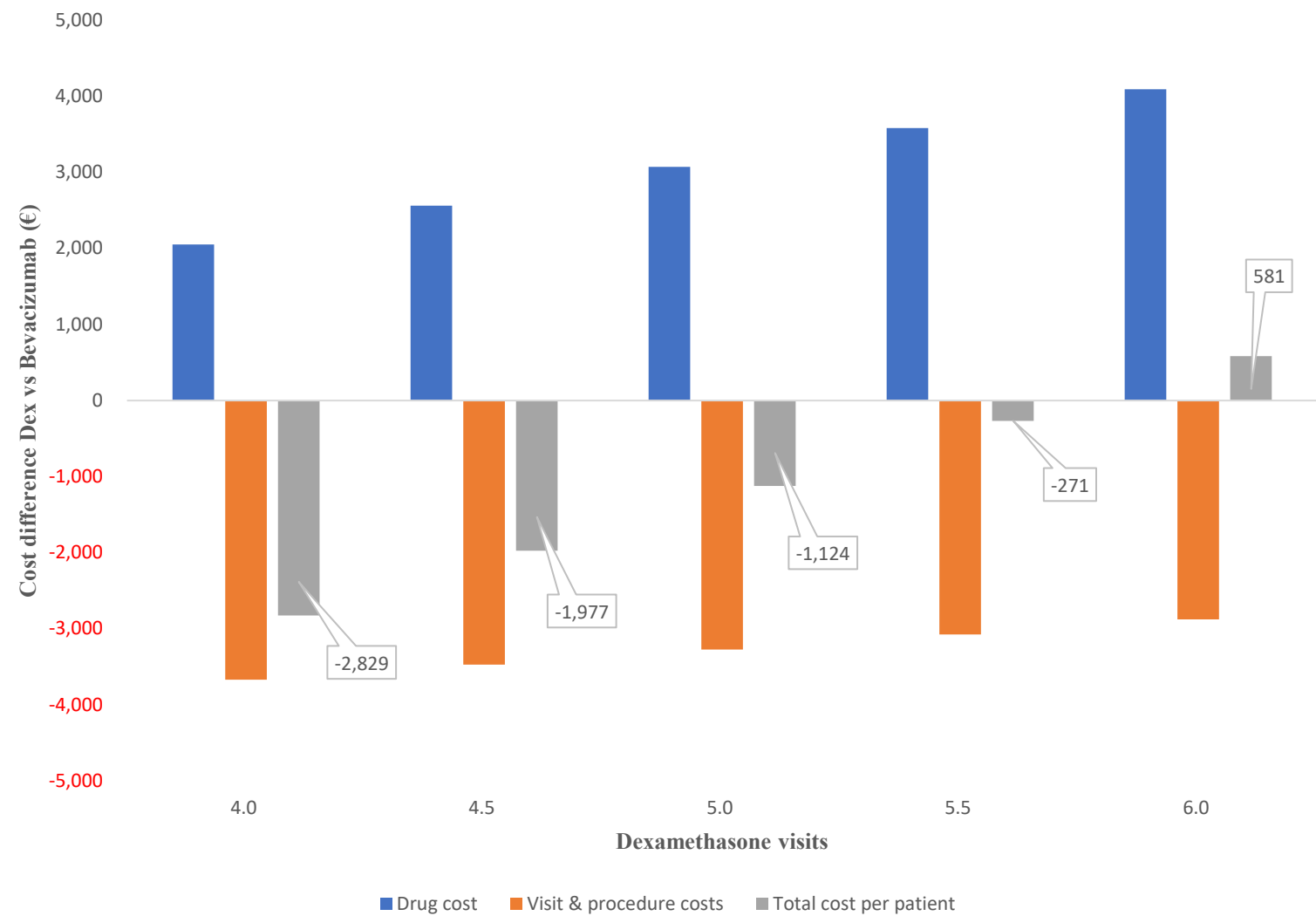

Supplement: Supplementary file 2 — Figure S2. One‐way sensitivity analysis for the two‐year cost differences per patient for dexamethasone (DEX) compared with bevacizumab by the number of dexamethasone visits for Naive patients in the ‘extended’ healthcare perspective. [file AOS-100-e1630-s003.pdf]
