# Supplementary material for: Healthcare expenditure of intravitreal anti‐vascular endothelial growth factor inhibitors compared with dexamethasone implant for diabetic macular oedema
Source: Acta Ophthalmol. 2022 Apr 25;100(8):e1630–40. doi: 10.1111/aos.15151 (PMC9790387; doi:10.1111/aos.15151)
Supplement: Supplementary file 3 — Table S1. Treatment modalities and cost for diabetic macula oedema with the number of patients treated at the Oslo University Hospital in the Naive group (initiated on bevacizumab in 2016–2018). [file AOS-100-e1630-s001.docx]

**Supplementary Appendix**

To explore the potential variation in injection use between the first and second years, we collected additional follow-up data for Naïve patients (as defined in the Methods section). These registry data were collected for patients who initiated treatment in 2016 on bevacizumab and were followed through 2018: 119 such patients were identified from which 50 patients had a complete follow-up time of 24 months over 2 consecutive years.

We found a total of 818 injections for the 24 months of follow-up, with an average of 16.4 per patient over that period. When stratified by the first and the second 12 months of use, the first 12 months had on average 9.3 injections, while the subsequent 12 months had lower average or 7.0 injections per patient. (Supplementary Table S1).

Analysis of this data produced similar results to our previous findings for the initial year and our sensitivity analysis covered possible uncertainties in the follow-up years (main manuscript Table 3 & 5).

**Supplementary Table S1.** Treatment modalities and cost for Diabetic Macula Edema with the number of patients treated at the Oslo University Hospital in the Naive group (initiated on bevacizumab in 2016 - 2018)

|  |  |  | **Total** | **Average** |
| --- | --- | --- | --- | --- |
| **2016 patient years (N=50) -** | **First year** | **Injections** | 467 | 9.3 |
|  |  | **Cost** | € 536208 | € 1148 |
|  |  | **Bevacizumab** | 344 |  |
|  |  | **Aflibercept** | 118 |  |
|  |  | **Ranibizumab** | 5 |  |
|  | **Second year** | **Injections** | 351 | 7 |
|  |  | **Cost** | € 537188 | € 1530 |
|  |  | **Bevacizumab** | 213 |  |
|  |  | **Aflibercept** | 116 |  |
|  |  | **Ranibizumab** | 22 |  |

For the supplementary caculation we assumed that the 1st year, dexamethasone implant is used as in the manuscript (3), and the 2nd year to have reduced to an average of 2 dexamethasone implants, while for the entire period an average of 5 dexamethasone implants. We have further perfomed a sensintivity analysis on the uncertainties that may prevail. In **Figure S1** we varied both bevacizumab and DEX implant’s possible injections per year per patient and in **Figure S2** we held constant the average 2 year bevacizumab in the data and varied possible 2 year average DEX implant injections per patient.

**Supplementary** **Figure S1:** Two-way sensitivity analysis for the total two-year cost differences per patient for dexamethasone (DEX) compared to bevacizumab by the number of visits for both drugs in Naive patients in the ‘extended’ healthcare perspective

**Supplementary** **Figure S2:** One-way sensitivity analysis for the two-year cost differences per patient for dexamethasone (DEX) compared to bevacizumab by the number of dexamethasone visits for Naive patients in the ‘extended’ healthcare perspective
